# RETRACTION: Bach2 overexpression represses Th9 cell differentiation by suppressing IRF4 expression in systemic lupus erythematosus

**DOI:** 10.1002/2211-5463.13922

**Published:** 2024-11-01

## Abstract

**RETRACTION**: Y. Sheng, J. Zhang, K. Li, H. Wang, W. Wang, L. Wen, J. Gao, X. Tang, H. Tang, H. Huang, M. Cai, T. Yuan, L. Liu, X. Zheng, Z. Zhu and Y. Cui, “Bach2 Overexpression Represses Th9 Cell Differentiation by Suppressing IRF4 Expression in Systemic Lupus Erythematosus,” *FEBS Open Bio* 11, no. 2 (2021): 395–403, https://doi.org/10.1002/2211-5463.13050.

The above article, published online on 28 November 2020 in Wiley Online Library (wileyonlinelibrary.com), has been retracted by agreement between the authors; the journal Editor‐in‐Chief, Miguel A. De la Rosa; FEBS Press; and John Wiley and Sons Ltd. The retraction has been agreed following diagnostic errors identified by the authors in the patient samples used in this study. Based on the issues identified, the editors and authors consider the conclusions substantially compromised.

**RETRACTION**: Y. Sheng, J. Zhang, K. Li, H. Wang, W. Wang, L. Wen, J. Gao, X. Tang, H. Tang, H. Huang, M. Cai, T. Yuan, L. Liu, X. Zheng, Z. Zhu and Y. Cui, “Bach2 Overexpression Represses Th9 Cell Differentiation by Suppressing IRF4 Expression in Systemic Lupus Erythematosus,” *FEBS Open Bio* 11, no. 2 (2021): 395–403, https://doi.org/10.1002/2211-5463.13050.

The above article, published online on 28 November 2020 in Wiley Online Library (wileyonlinelibrary.com), has been retracted by agreement between the authors; the journal Editor‐in‐Chief, Miguel A. De la Rosa; FEBS Press; and John Wiley and Sons Ltd. The retraction has been agreed following diagnostic errors identified by the authors in the patient samples used in this study. Based on the issues identified, the editors and authors consider the conclusions substantially compromised.

